# Supplementary material for: Methamphetamine induces cardiomyopathy by Sigmar1 inhibition-dependent impairment of mitochondrial dynamics and function
Source: Commun Biol. 2020 Nov 17;3:682. doi: 10.1038/s42003-020-01408-z (PMC7673131; doi:10.1038/s42003-020-01408-z)

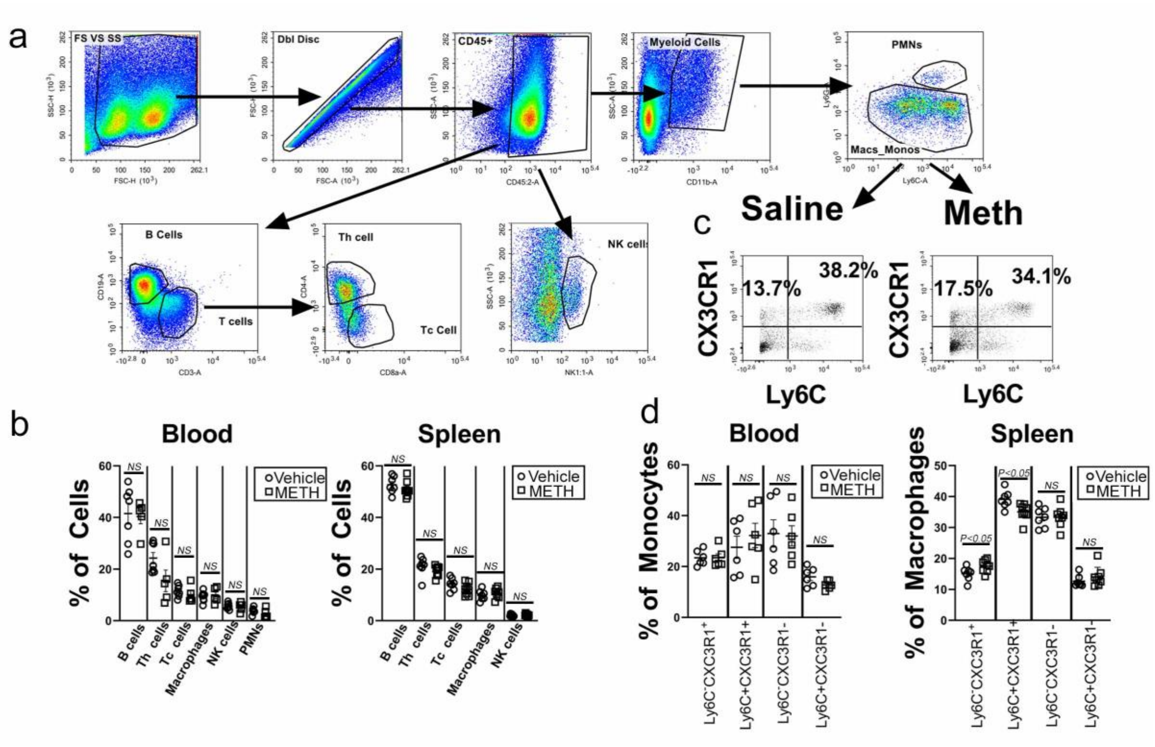

Supplementary Figure 1. Immunophenotyping in systemic circulation and secondary lymphoid organ spleen of Vehicle and METH treated mice. a Immune cells were analyzed through flow cytometry in live single-cell suspensions from blood and splenocytes. Representative forward and side scatter profiles showing gating strategy used to quantify the percent of immune cell populations in blood and spleen. **b** Left panel, bar graphs represent percent population of B cells ($CD45^+CD19^+$), Th cells ($CD45^+CD19^-CD3^+CD4^+$), Tc cells ($CD45^+CD19^-CD3^+CD8^+$), macrophages ($CD45^+CD11b^+Ly6G^-Ly6C^+$), natural killer (NK) cells ($CD45^+NK1.1^+$), and polymorphonuclear cells (PMNs) ($CD45^+CD11b^+Ly6G^+Ly6C^+$) in the blood of vehicle (n=6-7) and METH (n=5) treated mice. Right panel, bar graph represents the number of B Cells, Th cells, Tc cells, macrophages, and NK cells in spleen expressed as a percent of total splenocytes population in the vehicle (n=7) and METH (n=8) treated mice. **c-d** Bar graphs represent $CD45^+CD11b^+Ly6G^-Ly6C^+CX3Cr1^-$ and $CD45^+CD11b^+Ly6G^-Ly6C^+CX3Cr1^+$ monocytes expressed as a percent of total monocytes in the blood (n=6 mice per group) and spleen in the vehicle (n=7) and METH (n=8) treated mice, respectively. Data are expressed as mean ± SEM. *P* values were determined by two-tailed unpaired Student's t-test. A *P* value of less than 0.05 between groups considered statistically significant. METH, methamphetamine.

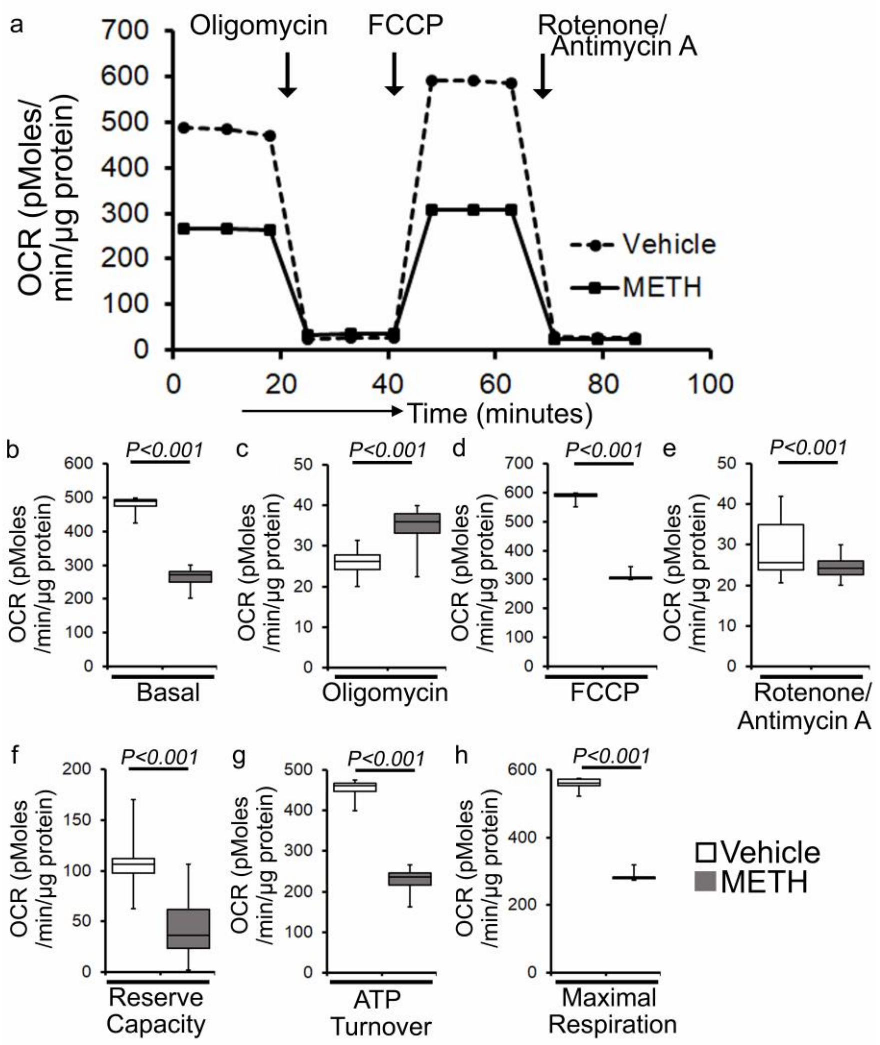

Supplementary Figure 2. METH treatment attenuates mitochondrial respiratory parameters in cardiac mitochondria. a Summary traces of mitochondrial oxygen consumption rate (OCR) values measured in isolated cardiac mitochondria from Vehicle and METH treated mice post-2-weeks of treatment. Black arrow indicates the sequential addition of oligomycin (1 µmol/L), carbonyl cyanide 4-(trifluoromethoxy) phenylhydrazone (FCCP) (4 µmol/L), and rotenone (0.5 µmol/L) plus antimycin A (0.5 µmol/L). OCR values are expressed as picomoles of O_2/[min*µg protein], and each point represents average OCR values from 6 individual mice per group. **b-e** Bar graphs represent OCR under (**b**) baseline as well as with the addition of (**c**) oligomycin, (**d**) FCCP, and (**e**) rotenone plus antimycin A. **f-h** Key parameters of mitochondrial bioenergetics, including (**f**) reserve capacity, (**g**) ATP turnover, and (**h**) maximal respiration, were significantly decreased in METH treated mice heart mitochondria compared to vehicle-treated group. Boxes depict interquartile ranges, lines represent medians, and whiskers represent ranges. *P* values were determined by Tukey's pairwise multiple comparisons test. A *P* value of less than 0.05 between groups considered statistically significant. Veh, vehicle; METH, methamphetamine.

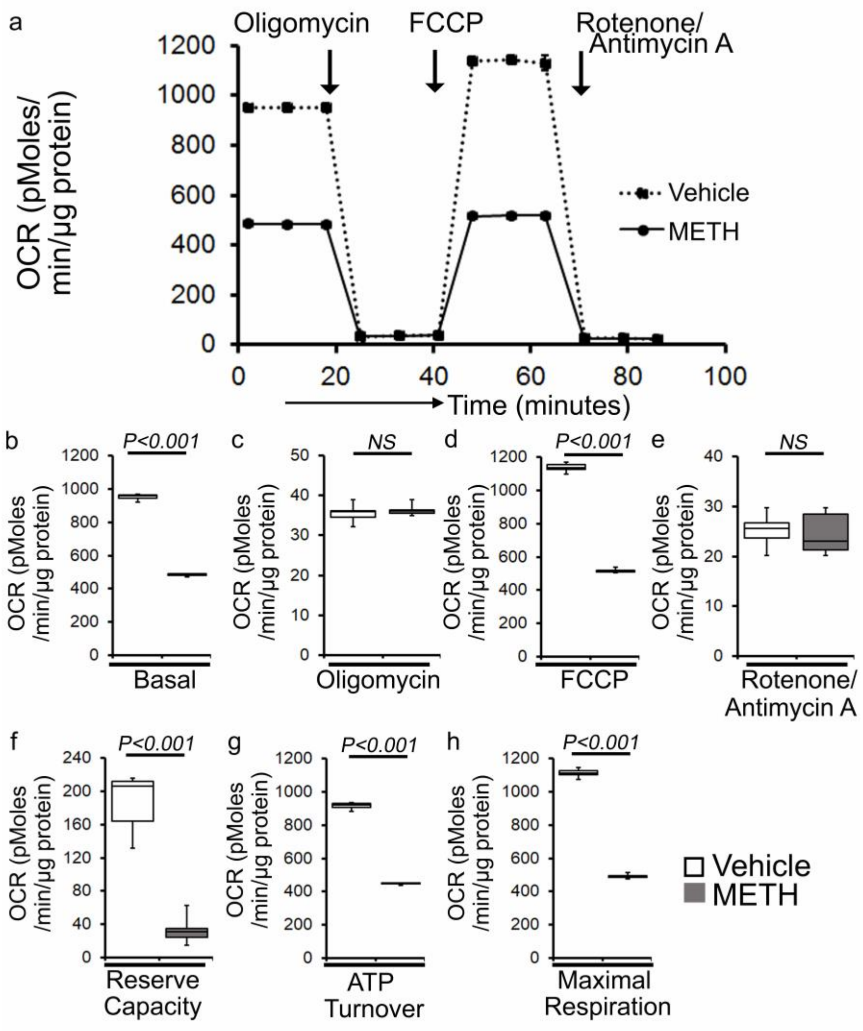

Supplementary Figure 3. METH induces suppression of mitochondrial respiration in cultured cardiomyocytes. a Summary traces of mitochondrial oxygen consumption rate (OCR) profiles measured in cultured neonatal rat cardiomyocytes (NRC) following treatment with vehicle or METH (100 µM) for 24 hours. The black arrow indicates the sequential addition of oligomycin (1 µmol/L), carbonyl cyanide 4-(trifluoromethoxy) phenylhydrazone (FCCP) (4 µmol/L), and rotenone (0.5 µmol/L) plus antimycin A (0.5 µmol/L). OCR profiles are expressed as pmol O_2/[min*µg protein], and each point represents average OCR values from 5 wells per group. **b-e** Bar graphs represent OCR under (**b**) baseline and with the addition of (**c**) oligomycin, (**d**) FCCP, and (**e**) rotenone plus antimycin A. (**f-h**) Key parameters of mitochondrial bioenergetics, including (**f**) reserve capacity, (**g**) ATP turnover, and (**h**) maximal respiration, were significantly decreased in METH-treated cardiomyocytes compared to those of the vehicle-treated group. Boxes depict interquartile ranges, lines represent medians, and whiskers represent ranges. *P* values were determined by Tukey's pairwise multiple comparisons test. $P<0.05$ between groups was considered statistically significant. Veh, vehicle; METH, methamphetamine.

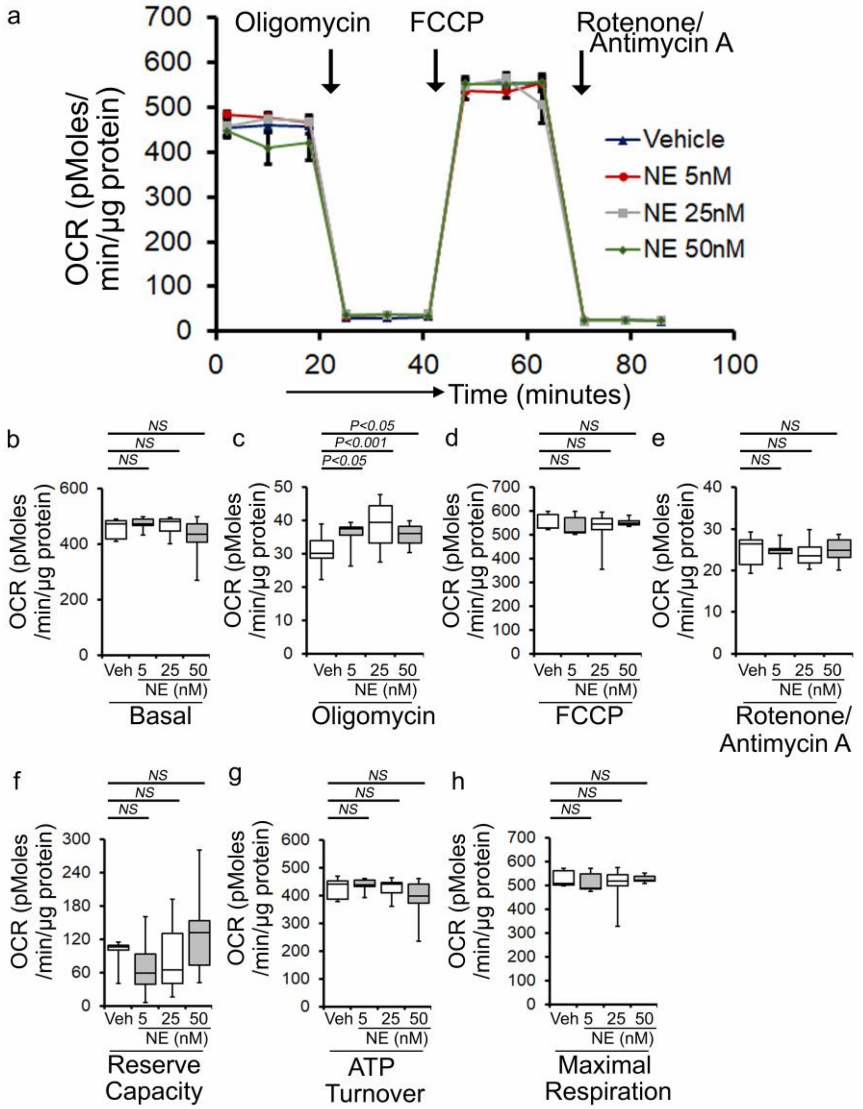

Supplementary Figure 4. Mitochondrial respiratory parameters in norepinephrine treated cultured cardiomyocytes. a Summary traces of mitochondrial oxygen consumption rate (OCR) profiles measured in cultured neonatal rat cardiomyocytes (NRCs) following treatment with vehicle (media only) and norepinephrine (NE) (5 nM, 25 nM and 50 nM) for 24 hours. Black arrow indicates the sequential addition of oligomycin (1 µmol/L), carbonyl cyanide 4-(trifluoromethoxy) phenylhydrazone (FCCP) (4 µmol/L), and rotenone (0.5 µmol/L) plus antimycin A (0.5 µmol/L). OCR profiles are expressed as picomoles of O_2/[min*µg protein], and each point represents average OCR values from 5 wells per group. **b-e** Bar graphs represent OCR under (**b**) baseline as well as with the addition of (**c**) oligomycin, (**d**) FCCP, and (**e**) rotenone plus antimycin A. **f-h** Key parameters of mitochondrial bioenergetics, including (**f**) reserve capacity, (**g**) ATP turnover, and (**h**) maximal respiration, were significantly decreased in METH treated cardiomyocytes compared to vehicle-treated group. Boxes depict interquartile ranges, lines represent medians, and whiskers represent ranges. *P* values were determined by one-way ANOVA followed by Tukey's pairwise multiple comparisons test. A *P* value of less than 0.05 between groups considered statistically significant. Veh, vehicle; METH, methamphetamine.

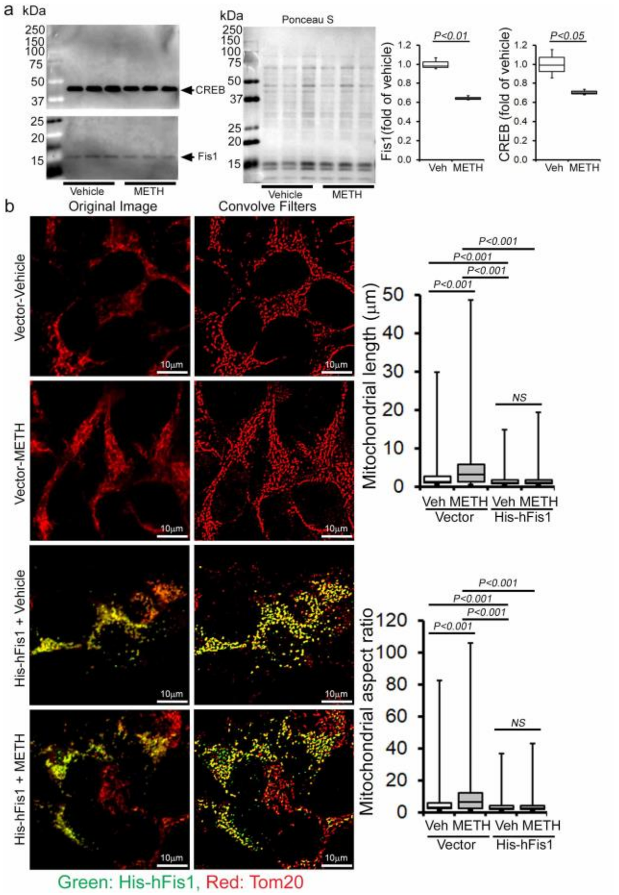

Supplementary Figure 5. Fis1 overexpression preserved METH-induced altered mitochondrial morphology. a Left panel, representative western blot images of CREB, and Fis1 expression following 24 hours of saline (vehicle) and METH (100 μM) treatment to HEK293T cells in triplicates. Right panel, bar graphs of Western blot lanes quantification showing METH reduces Fis1 and CREB proteins level in HEK293T cells. **b** Left panel, representative confocal microscope, and corresponding convolved images of Tom20 (in red to stain mitochondria) and His-tag (in green to stain exogenously overexpressed human Fis1 plasmid) stained HEK293T cells. Respective convolved images were used to analyze mitochondrial morphometric parameters (length and width) in vector-vehicle (6871 mitochondrial networks in 200 cells), vector-METH (2943 mitochondrial networks in 242 cells), His-hFis1+vehicle (4385 mitochondrial networks in 204 cells) and His-hFis1+METH (3831 mitochondrial networks in 201 cells) groups of HEK293T cells from three independent experiments. Right panel, bar graphs of measured mitochondrial length (μm) and mitochondrial aspect ratio (length-to-width ratio) confirming His-hFis1 overexpression induces mitochondrial fragmentation as evident in reduced mitochondrial length and aspect ratio. Moreover, His-hFis1 overexpression preserved METH-induced mitochondrial networks length elongation compared to vehicle only treated cells. Vehicle and METH group cells were transfected only with an empty vector plasmid. Boxes depict interquartile ranges, lines represent medians, and whiskers represent ranges. *P* values were determined by one-way ANOVA followed by Tukey's multiple comparisons test. A *P* value of less than 0.05 between groups considered statistically significant. Veh, vehicle; METH, methamphetamine; His-hFis1, N-terminal His-tagged human Fis1.

Sample ID	Cause of Death	Age & Sex	Heart Wt.	Body Wt.	BMI	Height	Race
METH-1	Stab wound	51y M	520 g	216 lbs	32.4	5'8"	C
METH-2	Acute cardiovascular disease	54y M	570 g	198 lbs	32	5'6"	C
METH-3	Cocaine/Methamphetamine toxicity	55y M	470 g	210 lbs	29.3	5'11"	AA
METH-4	Hanging (suicide)	26y M	460 g	211 lbs	29.4	5'11"	C
METH-5	Combined drug overdose	45y M	450 g	187 lbs	24.7	6'1"	C
METH-6	Combined drug overdose	50y F	320 g	139 lbs	27.1	5'0"	C
METH-7	Chronic myocardial infarction	30y F	365 g	198 lbs	31	5'7"	C
METH-8	Methamphetamine toxicity	20y F	270 g	124 lbs	21.3	5'4"	C
METH-9	Gunshot wound	31y M	310 g	154 lbs	21.5	5'11"	C
METH-10	Gunshot wound	28y M	340 g	167 lbs	22	6'3"	AA
METH-11	Methamphetamine toxicity	20y M	305 g	180 lbs	25.8	5'10"	C
METH-12	Gunshot wound	23y F	290 g	171 lbs	26.8	5'7"	C
METH-13	Stab wound	34y M	390 g	160 lbs	26.6	5'5"	C
METH-14	Blunt force injuries	34y M	320 g	182 lbs	30.3	5'5"	C
METH-15	Pulmonary emboli	50y M	430 g	228 lbs	30.1	6'1"	C
METH-16	Pulmonary emboli	36y F	500 g	331 lbs	51.8	5'7"	C
METH-17	Blunt force injuries	34y M	390 g	172 lbs	26.9	5'7"	C
METH-18	Blunt force injuries	28y F	360 g	205 lbs	30.3	5'9"	C
METH-19	Hanging (suicide)	28y M	370 g	157 lbs	21.9	5'11"	C
METH-20	Gunshot wound	45y M	510 g	291 lbs	41.7	5'10"	AA
METH-21	Methamphetamine toxicity	63y M	500 g	161 lbs	23.8	5'9"	C
METH-22	Gunshot wound	49y M	430 g	226 lbs	30.6	6'0"	C
METH-23	Gunshot wound	29y M	410 g	199 lbs	29.4	5'9"	AA
METH-24	Gunshot wound	45y M	400 g	173 lbs	27.1	5'7"	AA
METH-25	Gunshot wound	55y M	340 g	162 lbs	26.1	5'6"	C
METH-26	Gunshot wound	48y M	500 g	144 lbs	20.1	5'11"	C
METH-27	Blunt force injuries	33y M	400 g	221 lbs	30.8	5'11"	C
METH-28	Methamphetamine intoxication	36y M	350 g	167 lbs	24	5"10"	AA
METH-29	Gunshot wound	30y M	430 g	170 lbs	26.6	5'7"	AA
METH-30	Methamphetamine intoxication	45y M	560 g	176 lbs	26	5'9"	C
METH-31	Methamphetamine toxicity	46y M	360 g	128 lbs	20	5'7"	C
METH-32	Gunshot wound (hypertension and alcoholic)	38y M	510 g	216 lbs	30.1	5'11"	C
METH-33	Hypertensive cardiovascular disease	41y M	715 g	278 lbs	38.8	5'11"	C
METH-34	Gunshot to head (alcoholic)	23y F	200 g	116 lbs	20.5	5'3"	AA

Control-1	Gunshot wound - Control Heart	32y M	395 g	248 lbs	35.6	5'10"	AA
Control-2	Hanging - Control Heart	36y M	250 g	122 lbs	18.5	5'8"	C
Control-3	Motor vehicle collision (MVC) - Control Heart	34y F	270 g	114 lbs	17.1	5'9"	AA
Control-4	Bilateral pulmonary thromboemboli - Control Heart	28y F	300 g	189 lbs	29.6	5'7"	AA
Control-5	Hanging (suicide) - Control Heart	18y F	290 g	108 lbs	18.5	5'4"	C
Control-6	Blunt force injuries - Control Heart	19y F	235 g	129 lbs	21.5	5'5"	C
Control-7	Gunshot wound - Control Heart	29y M	330 g	148 lbs	21.2	5'11"	AA

AA = African American, C = Caucasian

Supplementary Table 1: METH user and non-METH user patient demographics and physiological data

Day	Time			
	10 am	12 pm	2 pm	4 pm
1	0	0	0	0
2	1	1	2	2
3	3	3	4	4
4	5	5	5	5
5	6	6	6	6
6	--	--	--	--
7	--	--	--	--
8	0	0	0	0
9	3	3	4	4
10	5	5	5	5
11	6	6	6	6
12	6	6	6	6
13	--	--	--	--
14	--	--	--	--
15	0	0	0	0
16	3	3	4	4
17	5	5	5	5
18	6	6	6	6
19	6	6	6	6
20	--	--	--	--
21	--	--	--	--
22	0	0	0	0
23	3	3	4	4
24	5	5	5	5
25	6	6	6	6
26	6	6	6	6

Supplementary Table 2: Scheme for 'binge and crash' METH administration in mice. The dose of METH was escalated throughout the first cycle, which occurred during the first week of injections (days 1 through 5), followed by 3 weeks of repeated cycles of METH injections (days 8-12, 15-19, and 22-26) as shown in the table above. Mice received four injections per day two hours apart. The mice were injected at 10 am, 12 pm, 2 pm, and 4 pm with doses of METH including 0, 1, 2, 3, 4, 5, and 6 mg per kg body weight subcutaneously. Vehicle-treated mice received saline (0.9% w/v NaCl) in a scheme similar to that of METH treatment.

Uncropped Western Blots

Figure 5c

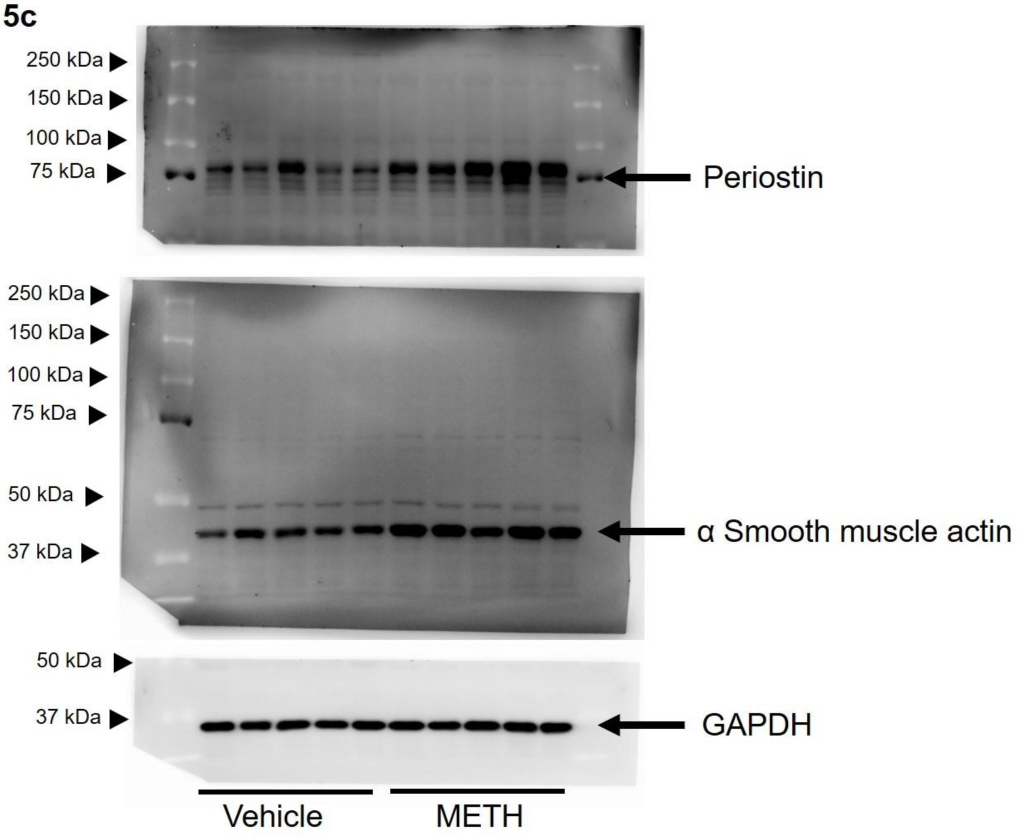

Figure 6a

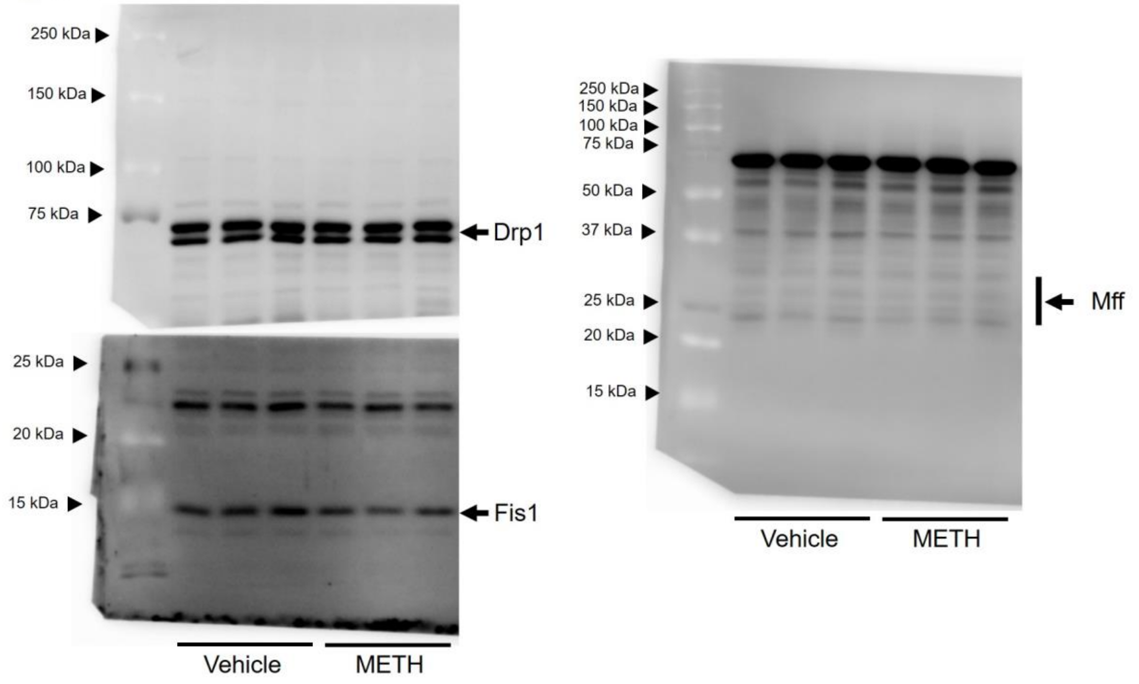

Figure 6a

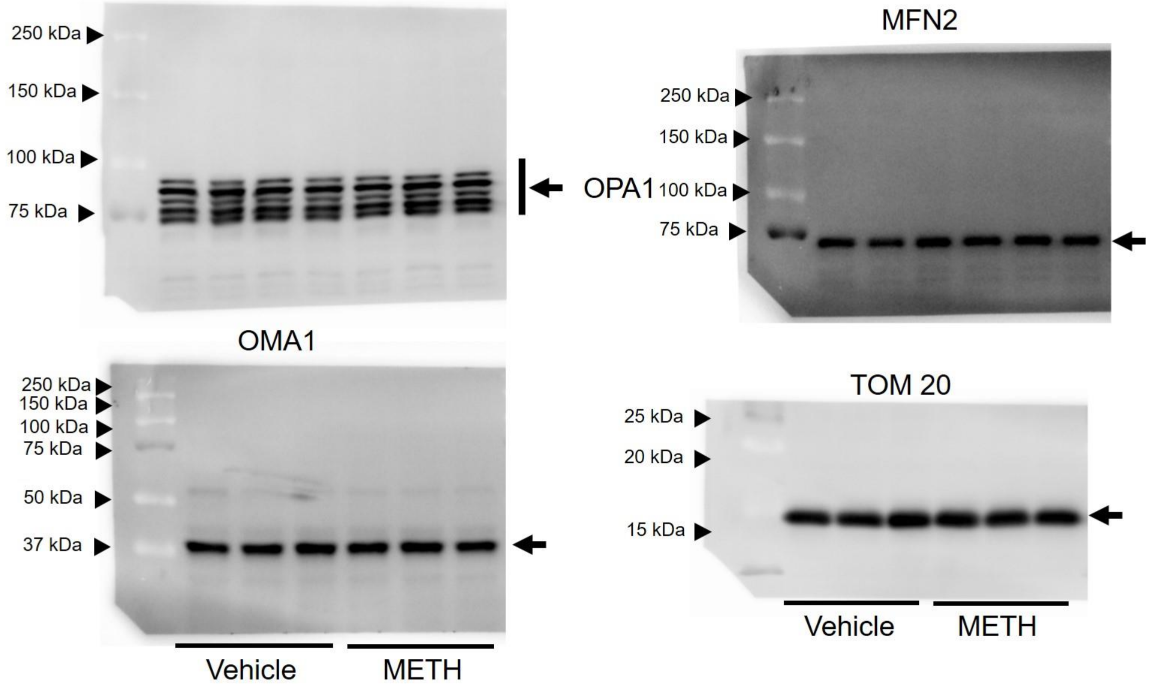

Figure 6a

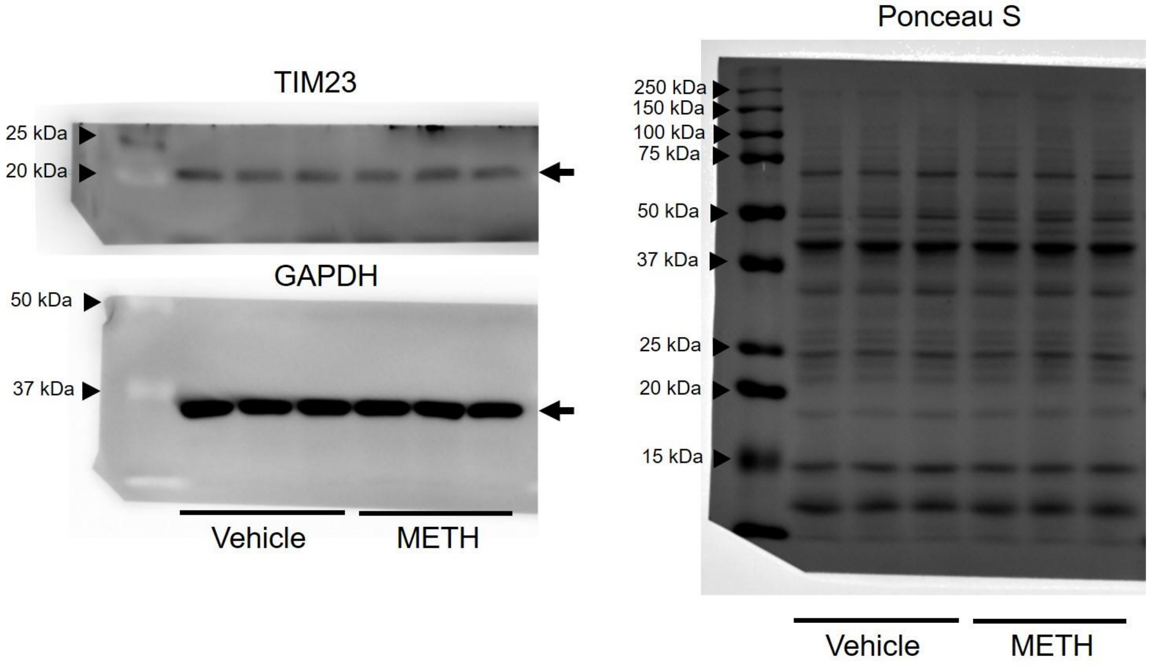

Figure 6b

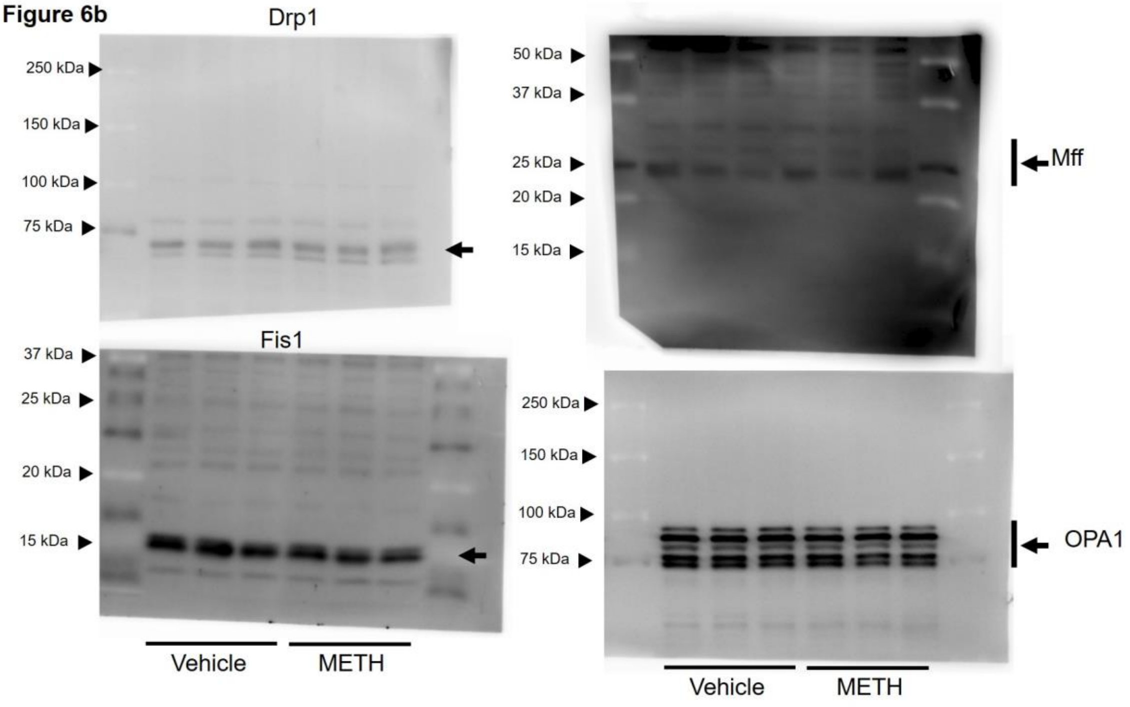

Figure 6b

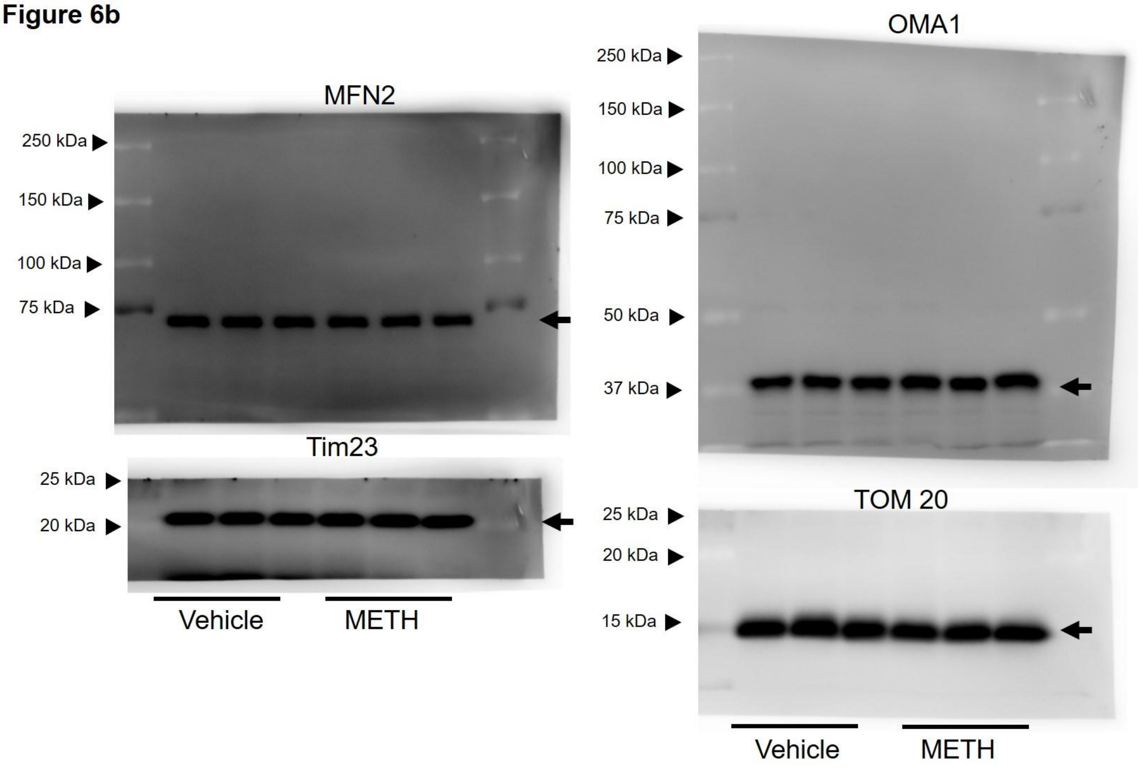

Figure 6b

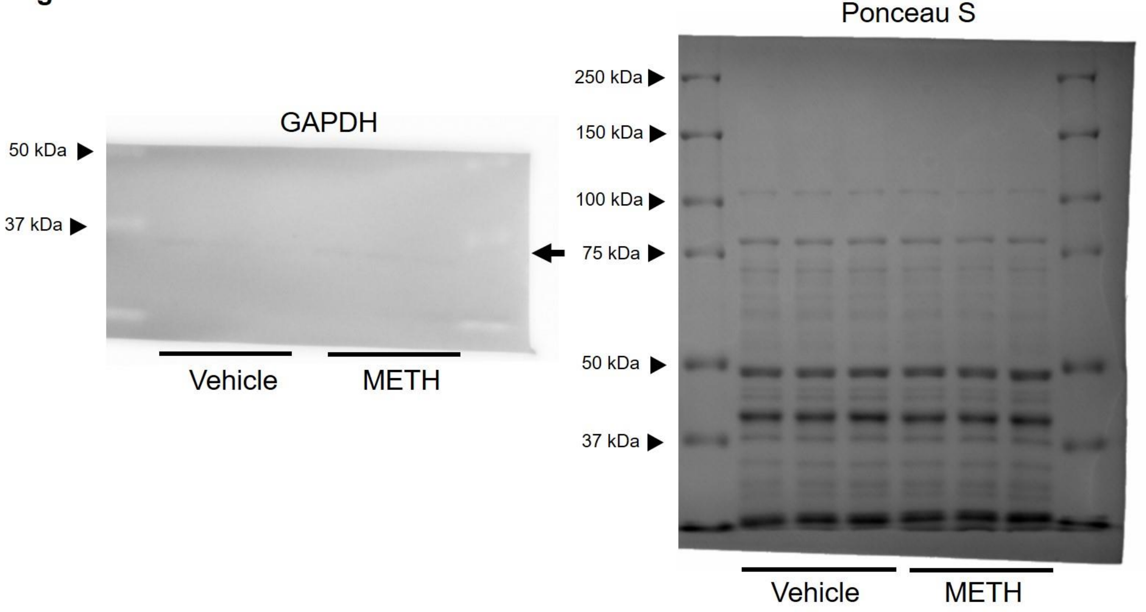

Figure 9c

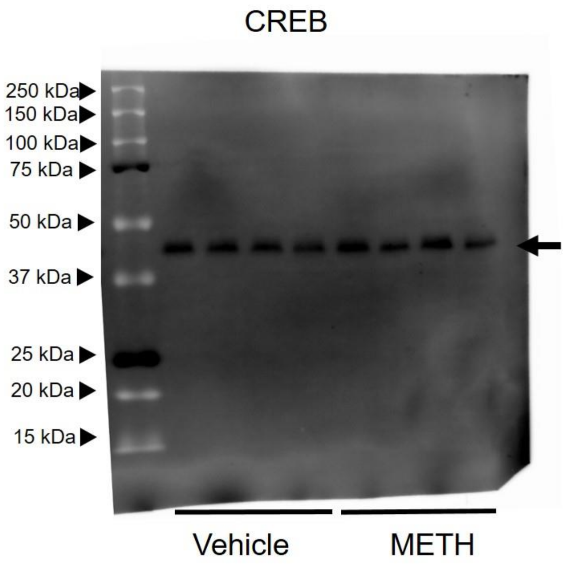

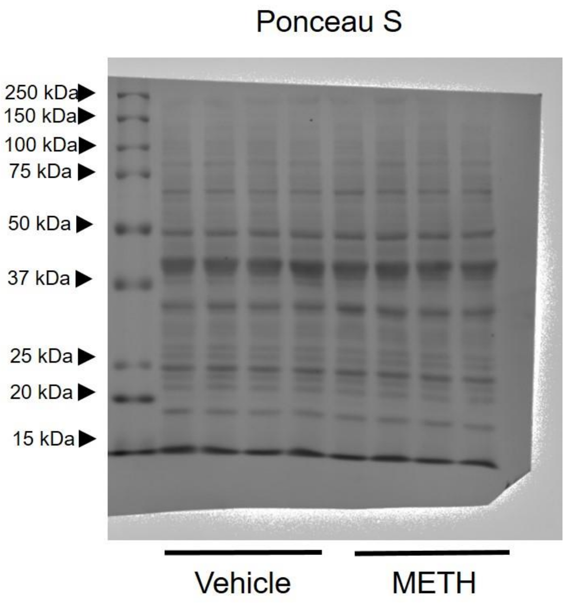

Figure 9c

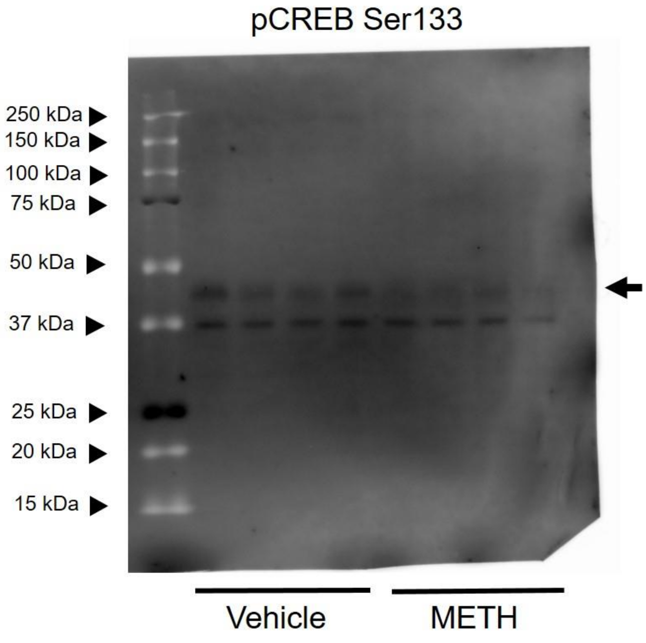

Figure 10d

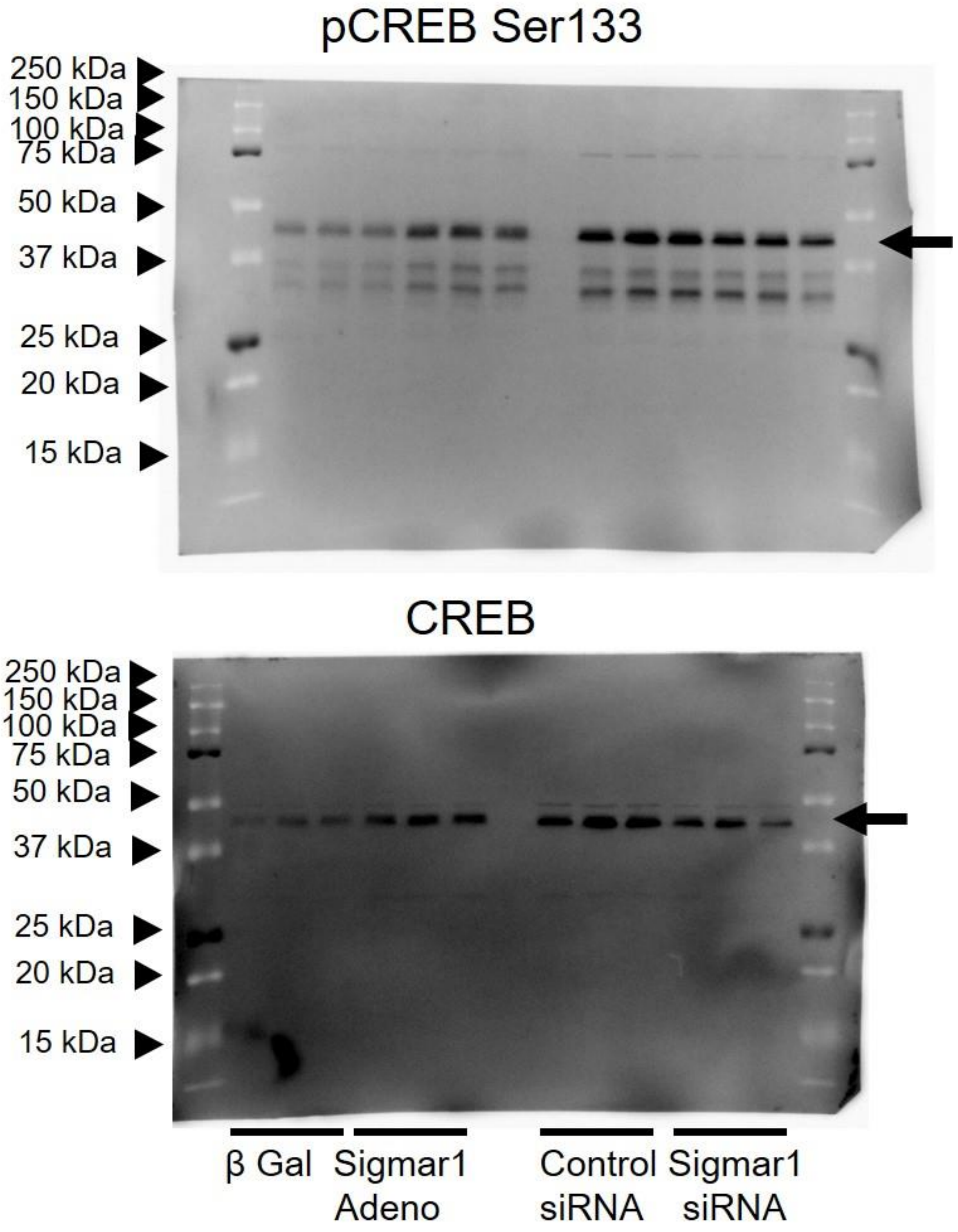

pCREB Ser133
250 kDa
150 kDa
100 kDa
75 kDa
50 kDa
37 kDa
25 kDa
20 kDa
15 kDa
CREB
250 kDa
150 kDa
100 kDa
75 kDa
50 kDa
37 kDa
25 kDa
20 kDa
15 kDa
β Gal
Sigmar1 Adeno
Control siRNA
Sigmar1 siRNA

Figure 10d

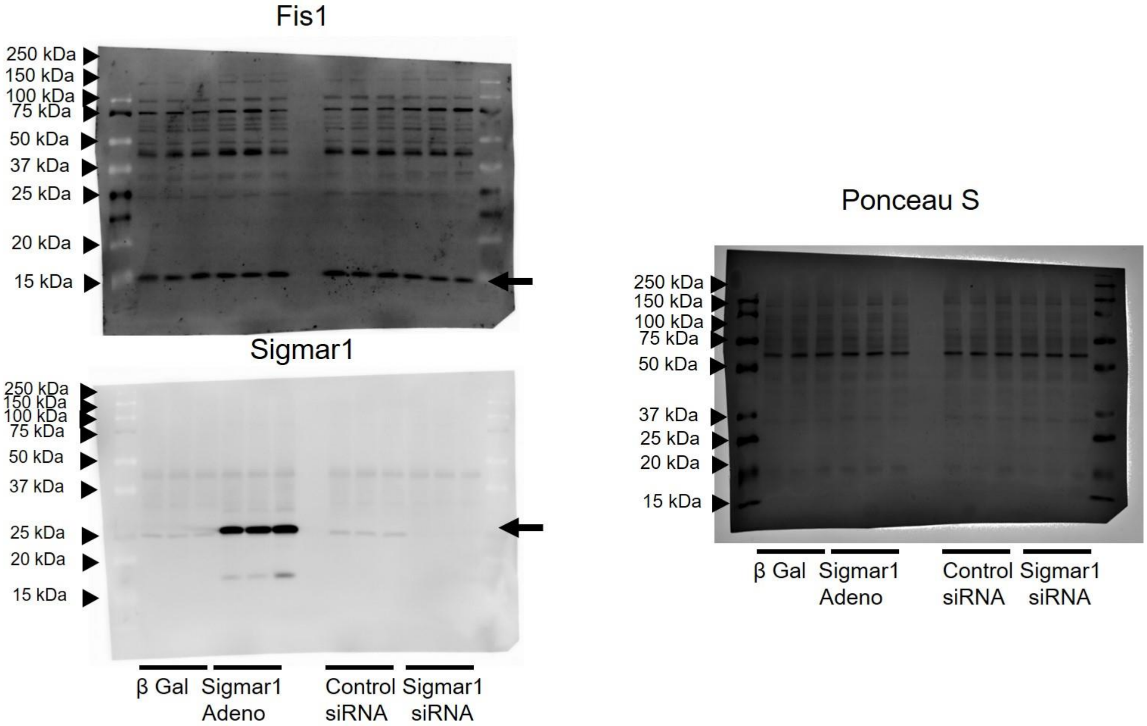
Fis1
250 kDa
150 kDa
100 kDa
75 kDa
50 kDa
37 kDa
25 kDa
20 kDa
15 kDa
Sigmar1
250 kDa
150 kDa
100 kDa
75 kDa
50 kDa
37 kDa
25 kDa
20 kDa
15 kDa
β Gal
Sigmar1 Adeno
Control siRNA
Sigmar1 siRNA
Ponceau S
250 kDa
150 kDa
100 kDa
75 kDa
50 kDa
37 kDa
25 kDa
20 kDa
15 kDa
β Gal
Sigmar1 Adeno
Control siRNA
Sigmar1 siRNA

Supplementary Figure 5a

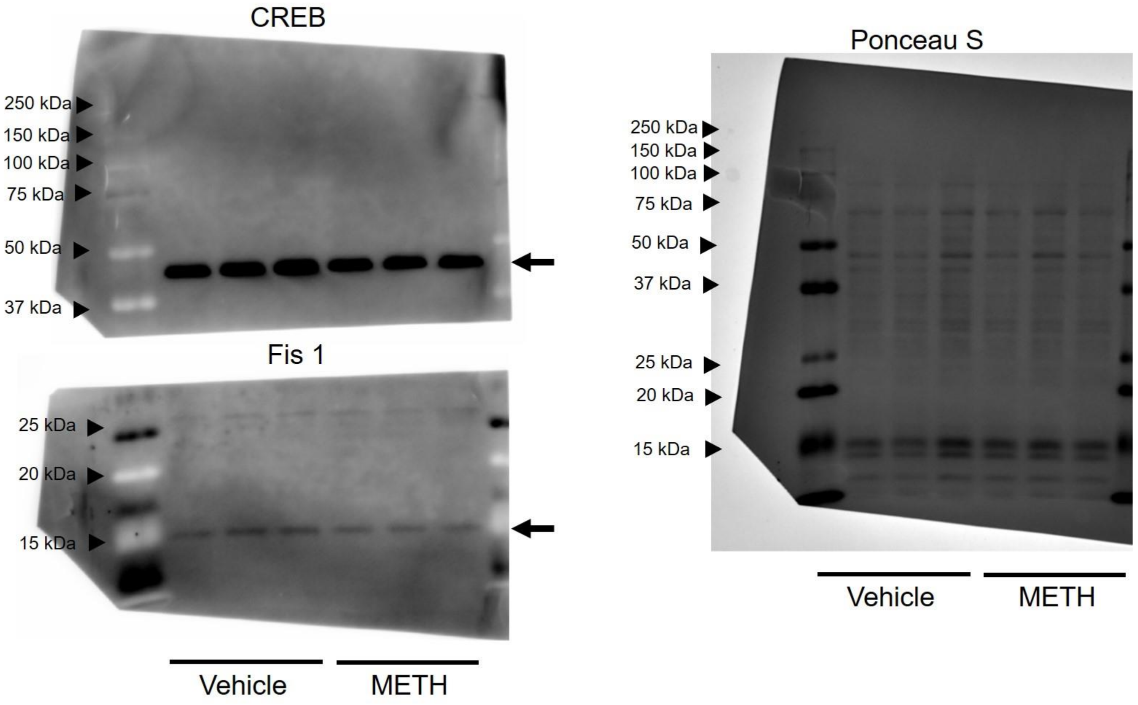

Supplement: Supplementary file 1 — Supplementary Information [file 42003_2020_1408_MOESM1_ESM.pdf]
