# Supplementary material for: Methamphetamine induces cardiomyopathy by Sigmar1 inhibition-dependent impairment of mitochondrial dynamics and function
Source: Commun Biol. 2020 Nov 17;3:682. doi: 10.1038/s42003-020-01408-z (PMC7673131; doi:10.1038/s42003-020-01408-z)
Supplement: Supplementary file 3 — Description of Additional Supplementary Files [file 42003_2020_1408_MOESM3_ESM.docx]

**Description of Additional Supplementary Files**

**Supplementary Data 1:** Source data underlying plots shown in figures.
