# Supplementary material for: Methamphetamine induces cardiomyopathy by Sigmar1 inhibition-dependent impairment of mitochondrial dynamics and function
Source: Commun Biol. 2020 Nov 17;3:682. doi: 10.1038/s42003-020-01408-z (PMC7673131; doi:10.1038/s42003-020-01408-z)
Supplement: Supplementary file 5 — Reporting Summary [file 42003_2020_1408_MOESM5_ESM.pdf]

nature research

Corresponding author(s): Md. Shenuarin Bhuiyan

Last updated by author(s): 2020/10/15

Reporting Summary

Nature Research wishes to improve the reproducibility of the work that we publish. This form provides structure for consistency and transparency in reporting. For further information on Nature Research policies, see Authors & Referees and the Editorial Policy Checklist.

Statistics

For all statistical analyses, confirm that the following items are present in the figure legend, table legend, main text, or Methods section.

n/a	Confirmed	
☐	☒	The exact sample size (n) for each experimental group/condition, given as a discrete number and unit of measurement
☐	☒	A statement on whether measurements were taken from distinct samples or whether the same sample was measured repeatedly
☐	☒	The statistical test(s) used AND whether they are one- or two-sided<br>*Only common tests should be described solely by name; describe more complex techniques in the Methods section.*
☐	☒	A description of all covariates tested
☐	☒	A description of any assumptions or corrections, such as tests of normality and adjustment for multiple comparisons
☐	☒	A full description of the statistical parameters including central tendency (e.g. means) or other basic estimates (e.g. regression coefficient) AND variation (e.g. standard deviation) or associated estimates of uncertainty (e.g. confidence intervals)
☒	☐	For null hypothesis testing, the test statistic (e.g. F, t, r) with confidence intervals, effect sizes, degrees of freedom and P value noted<br>*Give P values as exact values whenever suitable.*
☒	☐	For Bayesian analysis, information on the choice of priors and Markov chain Monte Carlo settings
☒	☐	For hierarchical and complex designs, identification of the appropriate level for tests and full reporting of outcomes
☒	☐	Estimates of effect sizes (e.g. Cohen's d, Pearson's r), indicating how they were calculated

Our web collection on statistics for biologists contains articles on many of the points above.

Software and code

Policy information about availability of computer code

Data collection	As detailed in the manuscript. We used standard, widely available instruments and softwares used in the field for our experimental data collection.
Data analysis	As detailed in the manuscript. We used standard, widely available softwares for data analysis in our studies.

For manuscripts utilizing custom algorithms or software that are central to the research but not yet described in published literature, software must be made available to editors/reviewers. We strongly encourage code deposition in a community repository (e.g. GitHub). See the Nature Research guidelines for submitting code & software for further information.

Data

Policy information about availability of data

All manuscripts must include a data availability statement. This statement should provide the following information, where applicable:

- Accession codes, unique identifiers, or web links for publicly available datasets
- A list of figures that have associated raw data
- A description of any restrictions on data availability

As given in the manuscript and supplementary information files.

Field-specific reporting

Please select the one below that is the best fit for your research. If you are not sure, read the appropriate sections before making your selection.

☒ Life sciences ☐ Behavioural & social sciences ☐ Ecological, evolutionary & environmental sciences

Life sciences study design

All studies must disclose on these points even when the disclosure is negative.

Sample size	No sample size calculation was performed. However, our sample sizes (animal numbers and biological replicates) for all studies were determined according to standards reported in the field by us and by others (for example, PMID: 30371279, 29987122, 30765730, 24177425, 27568194, 30891941)
Data exclusions	No samples or animals were excluded from the study.
Replication	All experiments were replicated multiple times with reproducible results as indicated in each figure legend.
Randomization	All animals were randomly allocated to each experimental groups.
Blinding	All experiments were performed in blinded fashion as detailed in the methods section of the manuscript and figure legends.

Reporting for specific materials, systems and methods

We require information from authors about some types of materials, experimental systems and methods used in many studies. Here, indicate whether each material, system or method listed is relevant to your study. If you are not sure if a list item applies to your research, read the appropriate section before selecting a response.

Materials & experimental systems

n/a	Involved in the study	
☐	☒	Antibodies
☐	☒	Eukaryotic cell lines
☒	☐	Palaeontology
☐	☒	Animals and other organisms
☒	☐	Human research participants
☒	☐	Clinical data

Methods

n/a	Involved in the study	
☒	☐	ChIP-seq
☐	☒	Flow cytometry
☒	☐	MRI-based neuroimaging

Antibodies

Antibodies used	Detailed information is provided in the methods section of the manuscript.
Validation	All used antibodies produced bands at the desired molecular weight and have been previously validated by the manufacturer: Drp1 (https://www.cellsignal.com/products/primary-antibodies/drp1-4e11b11-mouse-mab/14647), Fis1 https://www.scbt.com/p/fis1-antibody-fl-152), Mff (https://www.scbt.com/p/mff-antibody-b-2), OPA1 (https://www.cellsignal.com/products/primary-antibodies/opa1-d6u6n-rabbit-mab/80471), Mfn2 (https://www.cellsignal.com/products/primary-antibodies/mitofusin-2-d2d10-rabbit-mab/9482), Tom20 (https://www.scbt.com/p/tom20-antibody-fl-145), Tim23 (https://www.scbt.com/p/tim23-antibody-h-8), CREB (https://www.cellsignal.com/products/primary-antibodies/creb-48h2-rabbit-mab/9197), pCREBSer133 (https://www.cellsignal.com/products/primary-antibodies/phospho-creb-ser133-87g3-rabbit-mab/9198), Sigmar1 (https://www.cellsignal.com/products/primary-antibodies/sigmar1-d4j2e-rabbit-mab/61994), Tom20 (https://www.abcam.com/tomm20-antibody-mitochondrial-marker-ab56783.html), His (https://www.bethyl.com/product/A190-113A), GAPDH (https://www.emdmillipore.com/US/en/product/Anti-Glyceraldehyde-3-Phosphate-Dehydrogenase-Antibody-clone-6C5,MM_NF-MAB374).

Eukaryotic cell lines

Policy information about cell lines

Cell line source(s)	We used freshly isolated neonatal rat ventricular cardiomyocytes from 1-2 day old Sprague-Dawley rat pups as described in the methods section of the manuscript.
Authentication	The procedure and purity of isolation has all been standardized as reported in our earlier reports (for example, PMID: 22982234, 29987122, 30765730, 28667101). Further, we used sarcomeric associated protein Troponin I staining to confirm cardiomyocytes as provided in the manuscript results section.
Mycoplasma contamination	We used freshly isolated primary neonatal rat ventricular cardiomyocytes in culture media supplemented with 1x antibacterial-antimycotic (15240112, Gibco) containing Penicillin (100 units/mL), Streptomycin (100 ug/mL) and Amphotericin B (0.25 ug/mL) to prevent bacterial and fungal growth. We did not passage the cardiomyocytes culture, thus, have not coducted mycoplasma contamination test.
Commonly misidentified lines (See ICLAC register)	None.

Animals and other organisms

Policy information about studies involving animals; ARRIVE guidelines recommended for reporting animal research

Laboratory animals	As detailed in the manuscript.
Wild animals	N/A
Field-collected samples	N/A
Ethics oversight	Institutional Animal Care and Use Committee (ACUC) of Louisiana State University Health Sciences Center-Shreveport.

Note that full information on the approval of the study protocol must also be provided in the manuscript.

Human research participants

Policy information about studies involving human research participants

Population characteristics	As detailed in the manuscript Supplementary Table 1.
Recruitment	Patients were not specifically recruited for this study. All de-identified alpha-numerically labeled human left ventricular hearts sections and toxicology reports were postmortem autopsy heart samples in formalin jars collected in collaboration with pathologist Dr. James Traylor in Louisiana State University Health Sciences Center-Shreveport.
Ethics oversight	Louisiana State University Health Sciences Center-Shreveport.

Note that full information on the approval of the study protocol must also be provided in the manuscript.

Flow Cytometry

Plots

Confirm that:

- [X] The axis labels state the marker and fluorochrome used (e.g. CD4-FITC).
- [X] The axis scales are clearly visible. Include numbers along axes only for bottom left plot of group (a 'group' is an analysis of identical markers).
- [ ] All plots are contour plots with outliers or pseudocolor plots.
- [X] A numerical value for number of cells or percentage (with statistics) is provided.

Methodology

Sample preparation	As described in Supplementary Methods of the manuscript.
Instrument	As described in Supplementary Methods of the manuscript.
Software	As described in Supplementary Methods of the manuscript.
Cell population abundance	Expressed as percent of total cells.
Gating strategy	All conjugated antibodies were first titrated and compensated with compensation beads. Then, multi-color flow cytometry panel were constructed using fluorescence minus one control (FMOC) for all conjugated antibodies.

- [X] Tick this box to confirm that a figure exemplifying the gating strategy is provided in the Supplementary Information.
